# 非小细胞肺癌淋巴结微转移的研究进展

**DOI:** 10.3779/j.issn.1009-3419.2018.07.08

**Published:** 2018-07-20

**Authors:** 裕欢 赵, 东来 陈, 勇兵 陈

**Affiliations:** 1 215000 苏州，苏州大学附属第二医院胸心外科 Department of Cardiothoracic Surgery, the Second Affiliated Hospital of Soochow University, Suzhou 215000, China; 2 200433 上海，同济大学附属上海市肺科医院胸外科 Department of Thoracic Surgery, Shanghai Pulmonary Hospital, Tongji University School of Medicine, Shanghai 200433, China

**Keywords:** 肺肿瘤, 淋巴结, 微转移, Lung neoplasms, Lymph node, Micrometastasis

## Abstract

肺癌在我国是发病率和死亡率最高的恶性肿瘤，肿瘤发生转移是产生这种现象的重要因素。随着科学技术的发展，外科治疗对肺癌的效果有所提高。与此同时，分子靶向治疗的问世使肺癌的治疗达到了一个新的高度。但是，即使肿瘤在早期得到了完整切除，术后复发的可能性仍较高，这可能与肿瘤发生了淋巴结微转移（micrometastasis）有关。微转移逐渐成为现代医学研究的热点之一，相关的研究逐渐深入，但淋巴结微转移对肺癌手术方式的选择、病理分期及预后等方面的影响尚存在争议。本文综合论述了非小细胞肺癌（non-small cell lung cancer, NSCLC）淋巴结微转移的研究进展。

## Abstract (en)

The incidence and mortality of lung cancer rank top in China. One important factor is the occurrence of metastasis. With the development of science technology, the effect of surgical treatment on lung cancer is improved. Moreover, the use of targeted therapy has achieved a new height for the treatment of lung cancer. However, the recurrence rate remains high even the tumor was completely resected at early stage. The occurrence of lymph node micrometastasis is considered as one of the plausible explanations. The difficulty indetecting micrometastasis has been greatly reduced. Although studies dig deeper into the lymph node micrometastasis, there are still some controversies including the selection of surgical procedures, the pathological staging and prognosis about patients with lymph node micrometastasis. This review manages to generalize the latest research progress of lymph node micrometastasis.

非小细胞肺癌（non-small cell lung cancer, NSCLC）约占肺癌的80%，随着现代医疗技术的迅速发展，晚期NSCLC的5年生存率仍旧无法突破20%。即使是肿瘤完整切除的Ⅰ期NSCLC患者，术后复发或转移的几率仍然高达25%-30%^[1, 2]^，这些患者有可能早期就发生了淋巴结微转移（micrometastasis）。微转移已成为现代医学研究的热点之一^[3-5]^。近几年，随着分子生物学技术的空前进展，对微转移的研究进入了分子水平，这使得更快、更精确地检测微转移成为可能。

## 1 微转移的定义

肿瘤的微转移，又称隐匿性转移，即癌细胞扩散至全身各个组织、器官，但尚未形成显性的肿瘤结节，也未产生临床症状。在临床上，常规病理方法或计算机断层扫描（computed tomography, CT）等影像学检查经常用于检测肿瘤转移，但却无法检测出骨髓、外周血、淋巴结中的微转移^[6]^。

在淋巴结中检测到单个肿瘤细胞或细胞簇的最大直径 < 0.2 mm即淋巴结微转移^[7]^。这就为肺癌的术后复发提供了一种新的解释。由于评估术中清除淋巴结的技术的限制，如被正常细胞掩盖，小簇的肿瘤细胞容易被遗漏。因此，需要运用分子生物学技术进行更加完善的检测。

## 2 微转移的检测方法及标记物

检测微转移的最常用方法有聚合酶链式反应（polymerase chain reaction, PCR）和免疫组织化学染色（immunehistochemistry, IHC）。①PCR可作为检测淋巴结微转移的标准方法之一。PCR是大量扩增微量目的DNA片段的方法。它具有高度特异性、敏感性、高产率、可重复性等优点，因此在分子生物学研究中，PCR迅速地得到了广泛的应用。逆转录聚合酶链反应（reverse transcription PCR, RT-PCR）是目前较为常用的检测方法。RT-PCR是目前分析已知序列的RNA以及获取目的基因的最有效方法之一。RT-PCR技术检测肿瘤细胞的灵敏度可高达10^-5^-10^-7^。但其缺点是RNA在外周环境中易被污染，因而可能会产生一定的误差。PCR常用的标记物有肺组织特异性蛋白（lung-specific X protein, LUNX）、粘蛋白-1（mucin 1, MUC1）和端粒酶逆转录酶（human telomerase reverse transcriptase, hTERT）等。②IHC是检测淋巴结微转移的经典方法，因其方法相对简便、精确度高的优点受到了广泛应用，但是缺点是特异性较低。IHC是基于抗原抗体反应的检测方法，使用标记的抗体在组织或细胞对目标抗原进行定位、定性、定量检测。随着路德维希癌症研究所^[8]^和皇家马斯登医院的先导研究，运用IHC检测微转移开始兴起，在肺癌领域也有大量研究^[9]^。IHC常用的标记物有细胞角蛋白（cytokeratin, CK）、细胞角蛋白抗体AE1/AE3及Ber-EP4等。CKs广泛分布于上皮细胞源性肿瘤，因而CK抗体可检测不同类型癌症的隐匿性转移^[10, 11]^。然而，CKs不仅表达于肿瘤细胞，而且在正常上皮细胞也有表达，这就意味着在非肿瘤细胞中可能会出现假阳性。AE1/AE3抗体是AE1单克隆抗体与AE3单克隆抗体的混合物，两者可分别识别细胞角蛋白Ⅰ和细胞角蛋白Ⅱ。Dai等^[12]^选取了235例Ⅰ期肺腺癌患者，用AE1/AE3作为标记物，通过IHC进行研究，结果有35例（15%）患者有淋巴结微转移。③此外，尚有流式细胞技术（flow cytometry, FCM）、Cellsearch检测系统、蛋白免疫印迹（Western blot）等检测方法（表 1）。

**1:** 微转移的检测方法及标记物 Detection methods and markers for micrometastasis

Detection method	Markers	Effects
PCR	LUNX, MUC1, hTERT	High sensitivity, high specificity
IHC	CK, AE1/AE3, Ber-EP4	Low specificity, convenient
FCM	-	Low sensitivity
Cellsearch	CD45, EpCAM	High sensitivity, high specificity, high cost
Western blot	-	High sensitivity, high specificity
PCR: polymerase chain reaction; LUNX: lung-specific X protein; MUC1: mucin 1; hTERT: human telomerase reverse transcriptase; IHC: immunehistochemistry; CK: cytokeratin; FCM: flow cytometry; EpCAM: epithelial cellular adhesion molecule.		

## 3 发生淋巴结微转移的危险因素

Zhang等^[13]^选取54例常规病理检查Ⅰ期NSCLC患者108枚淋巴结和10例肺良性病变患者20枚淋巴结进行IHC染色检测葡萄糖调节蛋白94（glucose-regulated protein 94, GRP94），结果GRP94阳性率分别为20.4%和0，说明GRP94与淋巴结微转移有一定的相关性。有研究^[14]^指出在无淋巴结转移的患者中，nm23表达阳性组淋巴结微转移检出率比阴性组高，证明nm23是发生淋巴结微转移的因素之一。上皮钙粘蛋白（epithelial cadherin, E-cad）具有抑制转移的作用，E-cad低表达有可能是发生微转移的因素之一。研究认为血管内皮生长因子C（vascular endothelial growth factor-C, VEGF-C）与肿瘤发生淋巴道转移有关。李军等^[15]^通过实验发现肺癌组织中VEGF-C的表达明显比正常肺组织中高（54.55% *vs* 14.55%），并指出发生淋巴结微转移的肺癌组织中VEGF-C的表达明显比无淋巴结微转移的肺癌组织中高，VEGF-C可能参与了淋巴结微转移的启动过程。Kazakydasan等^[16]^通过研究证明VEGF-C高表达在一定程度上促进了淋巴结微转移的发生。

淋巴结微转移还与肿瘤的大小有关。Veeramachaneni等^[17]^选取297个临床Ⅰ期肺癌病例，通过逻辑回归分析指出肿瘤组织直径每增大1 cm，淋巴结微转移的发生概率就增大约3倍。CT是目前诊断肺癌的重要手段之一。一些影像学征象存在时，如淋巴结增大、淋巴结边缘不规则强化、淋巴结中心低密度坏死等，检测出淋巴结微转移的几率更高。

2011年，国际肺癌研究协会（International Association For The Study of Lung Cancer, IASLC）、美国胸科学会（American Thoracic Society, ATS）和欧洲呼吸学会（European Respiratory Society, ERS）发布了以5种常规组织学成分为依据的新腺癌分型，以此来更加精确地评估预后^[18]^。有研究^[1]^显示，在5种分型中，以微乳头成分为主的腺癌类型复发率最高，生存率最低，而淋巴结微转移也在此类型中较多见。Kamiya等^[19]^根据微乳头状成分比例，将383例腺癌患者分成4组，研究表明，与无微乳头状成分组相比，微乳头状腺癌组的无病生存期（disease free survival, DFS）和总生存期（overall survival, OS）都更差。此外，尚未有证据显示肺腺癌与肺鳞癌中淋巴结微转移的检出率存在差异。

## 4 淋巴结微转移在临床上的作用

IASLC在2015年发布了第八版肺癌肿瘤-淋巴结-转移（tumor-node-metastasis, TNM）分期，更加细分了肺癌的分期标准。淋巴结转移广泛应用于TNM分期中，评估患者预后和指导临床诊疗。一些研究表明，淋巴结微转移同样可以作为一个独立的影响因素起指导作用。Deng等^[3]^指出，在NSCLC中，有淋巴结微转移的患者复发的风险是无淋巴结转移患者的两倍。有数据^[12]^显示淋巴结微转移患者的5年生存率为39%，总生存率为62%，低于无淋巴结转移患者的80%与88%。

### 4.1 淋巴结微转移对手术方式选择的影响

肺肿瘤切除伴纵隔淋巴结清扫术被认为是早期NSCLC患者的标准治疗方法。Ⅰ期NSCLC患者接受淋巴结清扫者相较于未清扫淋巴结者有着更高的总生存期和无病生存期。但是，完整清除纵隔淋巴结是否能提高生存率仍然存在争议。淋巴结的清扫方式目前有3种，包括系统性淋巴结清扫（systematic nodal dissection, SND）、淋巴结采样（lymph nodal sampling, LNS）和肺叶特异性淋巴结清扫（lobe-specific nodal dissection, L-SND）。而不同清扫方式的清扫范围尚无统一标准^[20, 21]^。

淋巴结的检测方法决定了癌症病理分期的质量，SND能够较为精确地为术后分期提供依据。SND本身是否能够提高生存率存在争议，有研究^[22, 23]^认为SND仅对分期有作用，而对患者术后的生存期并无太大的作用。但是有学者通过实验确认SND降低了局部复发的几率。淋巴结微转移的发生是其中一种原因，有理由推测，SND能清除隐匿性微转移，从而阻止局部复发和远处转移。L-SND的定义最初是在2006年由欧洲胸外科学会提出来的^[24]^。Meng等^[25]^指出，对于早期NSCLS患者的预后，L-SND并不劣于SND，两者均优于LNS。L-SND由于能带来更小的手术创伤并减轻患者痛苦而逐渐受到认可，但是L-SND在检测淋巴结微转移上较为困难。

两种淋巴结清扫方式可根据术中检测是否有淋巴结微转移来选择，依照淋巴引流规律，区域淋巴结微转移阳性时可采用SND，而淋巴结微转移阴性时则可采用L-SND，再一次证明检测淋巴结微转移的重要性。Keller等^[26]^研究表明SND对淋巴结N2检出率明显优于LNS。Melfi等^[27]^研究发现右肺下叶的淋巴结N2转移率最高，而SND可有效规避微转移及跳跃转移。据研究^[28, 29]^报道，术中清扫更多的淋巴结，对患者的预后更有利。淋巴结微转移在清扫淋巴结多的病例中检出率更高。因此，行亚肺叶切除术或肺叶切除术者可适当增加淋巴结清扫数目。LNS是仅依靠术中观察、触觉等主观感觉清扫可疑的淋巴结，其清扫淋巴结数量较少，因而淋巴结微转移的检出率较低。

### 4.2 淋巴结微转移与病理分期的关系

淋巴结微转移对病理分期有一定的意义。Izbicki等^[30]^选取了肿瘤完整切除的常规病理pN0和pN1期共93例NSCLC患者与36例pN2期NSCLC患者，并使用单克隆抗体Ber-Ep4作为标记物使用IHC检测淋巴结微转移。结果发现72例pN0患者中有20例（27.4%）检测出存在微转移，20例pN1患者中9例（45.0%）有微转移。无论pN0还是pN1患者，无微转移者均较微转移者DFS长。研究得出结论：有淋巴结微转移存在的pN0和pN1患者的生存期与pN2患者的生存期并无差别。Qiu等^[31]^对193枚NSCLC的淋巴结进行微转移检测，再根据结果重新进行TNM分期，得出结论：从Ⅰ期提升至Ⅱ期的患者有2例，从Ⅱ期提升至Ⅲ期的患者有6例。有研究^[32]^指出，肿瘤的T分期越晚，发生淋巴结微转移的几率越高，术后复发的几率也增高。目前的常规病理分期并未将淋巴结微转移考虑在内，对患者的疾病情况无法作出更精确地反映。但淋巴结微转移是否应作为病理分期的参考因素，目前尚未有定论。

### 4.3 淋巴结微转移对预后的影响

疾病累及淋巴结是影响OS、DFS、复发率（recurrence rate, RR）的因素之一。通过分析（表 2）发现，大量研究证明淋巴结微转移对NSCLC患者的预后会产生不利影响，仅有少数研究认为两者关系不明确。可见，目前的观点倾向于淋巴结微转移是发生不良预后的原因之一。

**2:** 近十年肺癌淋巴结微转移的研究汇总 Summary of the research in lymph node micrometastasis of lung cancer in recent ten years

Author	No. of cases	Method	Marker	Detection rate	Prognostic relevance
Rena *et al* (2007)^[33]^	87	IHC	AE1/AE3	2.70%	No significance
Yamashita *et al* (2010)^[34]^	117	IHC	cytokeratin	29.1%	OS↓
Rusch *et al* (2011)^[35]^	1, 047	IHC	cytokeratin	22.4%	DFS、OS↓，RR↑
Dai *et al* (2013)^[36]^	49	RT-PCR	FHIT	22.0%	DFS、OS↓，RR↑
Martin *et al* (2016)^[37]^	502	IHC	AE1/AE3	14.0%	No significance
Dai *et al* (2017)^[12]^	235	IHC	AE1/AE3	37.0%	DFS、OS↓，RR↑
OS: overall survival; DFS: disease free survival; RR: recurrence rate.					

## 5 展望

目前的临床实践证明，淋巴结微转移的临床重要性尚未得到充分重视，是否需要将淋巴结微转移作为肺癌临床分期的一个因素，有待进一步研究。标记物的种类繁多，但尚未有公认的理想标记物，因此，未来研究的一个重要方向就是发现一种敏感性、特异性高的分子标记物。提高淋巴结微转移的检测水平，对肿瘤分期、指导临床治疗和评估预后有重要作用。对于检测出淋巴结微转移的患者的下一步治疗有待讨论，存在淋巴结微转移的Ia期患者是否应在术后进行辅助化疗及化疗后是否对患者有益尚无定论。淋巴结微转移对预后的影响尚存在争议。术中冰冻病理对微转移检测的作用尚未有结论。行肺叶切除术和亚肺叶切除术对术后病理提示存在淋巴结微转移的Ia期肺癌治疗效果有待对比研究。淋巴结微转移对各病理类型肺癌产生的作用是否相似尚不明确。淋巴结微转移的出现是否遵循肺叶特异性淋巴引流规律，及其带来的对选择手术方式的影响尚有待更多研究成果。
